# Enhanced passive safety surveillance of high‐dose and standard‐dose quadrivalent inactivated split‐virion influenza vaccines in Germany and Finland during the influenza season 2021/22

**DOI:** 10.1111/irv.13071

**Published:** 2022-11-29

**Authors:** Sonja Gandhi‐Banga, Sophie Wague, Anju Shrestha, Olga Syrkina, Oxana Talanova, Markku Nissilä, Karl Stuff, Céline Monfredo

**Affiliations:** ^1^ Sanofi Toronto ON Canada; ^2^ Sanofi Lyon France; ^3^ Sanofi Swiftwater Pennsylvania USA; ^4^ Affiliation at the time of the study; current affiliation Regeneron Pharmaceuticals Basking Ridge New Jersey USA; ^5^ Terveystalo Biobank and Clinical Research Turku Finland; ^6^ Arztpraxis Dr. Stuff Donaueschingen Germany; ^7^ Global Biostatistical Sciences, Sanofi Marcy l'Etoile France

**Keywords:** Efluelda, IIV4, influenza vaccine, quadrivalent split‐virion inactivated influenza vaccine, seasonal influenza, Vaxigrip Tetra

## Abstract

**Background:**

Enhanced passive safety surveillance (EPSS) was conducted for quadrivalent inactivated split‐virion influenza vaccines (IIV4) in Germany (high dose [HD], aged ≥60 years) and in Finland (standard dose [SD], aged ≥6 months) at the beginning of the northern hemisphere 2021/22 influenza season. The primary objective was to assess adverse drug reactions (ADRs) occurring ≤7 days post‐vaccination.

**Methods:**

Vaccinees were issued vaccination cards (VC) and were encouraged to report ADRs via an electronic data collection system or by telephone. ADRs were assessed by frequency, time to onset, intensity and by age group. The vaccinee reporting rate (RR) was calculated as the number of vaccinees reporting ≥1 ADR divided by total vaccinees. Reactogenicity was compared with previous experiences with each vaccine.

**Results:**

Among 903 HD‐IIV4 vaccinees in Germany, 17 reported ≥1 ADR within ≤7 days post‐vaccination: RR, 1.88% (95% CI: 1.10, 3.00). Time to onset was known for 53/65 ADRs, all of which occurred ≤7 days post‐vaccination. In Germany, seven ADRs were reported that were not listed previously. Among the 1000 SD‐IIV4 vaccinees in Finland, 49 reported ≥1 ADR within ≤7 days post‐vaccination: RR, 4.90% (95% CI: 3.65, 6.43). Time to onset was known for 126/134 ADRs, of which 125 occurred ≤7 days post‐vaccination. In Finland, 21 ADRs were reported that were not listed previously. No ADRs reported during follow‐up were serious.

**Conclusions:**

The EPSS for HD‐IIV4 and for SD‐IIV4 in the 2021/22 influenza season did not suggest any clinically relevant changes in safety beyond what is known/expected for IIV4s.

## 1 INTRODUCTION

Influenza vaccines need to be updated each year to match the strains predicted by the World Health Organization to be circulating in the forthcoming influenza season. This presents specific challenges for the continued and timely monitoring of safety for new vaccines each season. Therefore, in 2014, the European Medicines Agency (EMA) introduced the requirement for manufacturers to conduct enhanced passive safety surveillance (EPSS).^1^ The main aim of EPSS is to enhance safety signal detection in a routine clinical care setting by facilitating spontaneous reporting of adverse drug reactions (ADRs), in near real‐time, following vaccine exposure in the relevant age groups. Thus, any potential clinically significant changes in the frequency and/or intensity of ADRs can be detected before the peak immunization period each year.^1^

EPSS combines routine surveillance with clinical services to encourage patients and healthcare professionals (HCPs) to report adverse events. Unlike active surveillance, ADRs are not solicited with EPSS, and there is no active follow‐up unless the individual reports an ADR and agrees to be contacted for further information. The ability of EPSS to improve ADR reporting rates (RRs) after vaccination has been shown in several studies.^2^, ^3^, ^4^, ^5^, ^6^

Here we report the EPSS during the northern hemisphere (NH)^7^ 2021/22 influenza season for two quadrivalent inactivated split‐virion influenza vaccines (IIV4s). In Germany, we assessed high‐dose (HD)‐IIV4 containing 60 μg per strain (Efluelda®, Sanofi).^8^ HD‐IIV4 is licensed for use in adults aged ≥60 years and was launched in Germany in 2021. In people aged ≥65 years, HD‐IIV3 vaccine versus standard dose (SD) IIV3 vaccine has been shown to reduce cases of influenza‐like illness, as well as influenza‐related hospitalization and pneumonia.^9^ The addition of another B strain to HD‐IIV3 vaccine to formulate the HD‐IIV4 vaccine did not alter immunogenicity or safety.^10^ In Finland, we assessed SD‐IIV4 containing 15 μg per strain (Vaxigrip Tetra®, Sanofi).^11^ SD‐IIV4 was first licensed in Europe in 2016 for use in individuals aged ≥3 years, and in 2017, this was expanded in some countries to include use in individuals aged ≥6 months.^11^ Clinical studies have shown that SD‐IIV4 vaccine is immunogenic in children, adults and older adults, and a large, randomized, placebo‐controlled trial of children aged 6–35 months demonstrated efficacy of 71.7% (95% confidence interval [CI]: 43.7, 86.9) against laboratory‐confirmed severe influenza illnesses due to vaccine‐similar strains.^11^, ^12^

The EPSS of HD‐IIV4 and SD‐IIV4 were conducted to detect relevant changes in the frequency or intensity of expected reactogenicity compared with previous experience with the IIV4s. In Germany and Finland, each EPSS included individuals who received one of the vaccines at the start of the NH 2021/22 influenza season, with safety data collected for up to 8 weeks post‐vaccination using an electronic data capture (EDC) system with telephone reporting as back‐up.

## 2 METHODS

### 2.1 Design

This EPSS study was conducted to assess suspected ADRs reported by individuals receiving HD‐IIV4 vaccine in Germany or SD‐IIV4 vaccine in Finland during the NH 2021/22 influenza season. The EPSS in Germany and Finland were aligned with the start of seasonal influenza vaccination at participating sites, and safety data were collected for up to 8 weeks after the first vaccination. In Germany, participants aged ≥60 years received HD‐IIV4 vaccine at 10 sites between 18 October and 2 December 2021, and safety data were collected until 15 December 2021, that is, 2 weeks after the last person was vaccinated. In Finland, participants aged ≥6 months received SD‐IIV4 vaccine between 12 October and 10 November 2021, and safety data were collected until 23 November 2021.

Vaccines were administered as per routine clinical practice in accordance with the product labelling. The study was conducted in accordance with the Declaration of Helsinki, Good Epidemiological Practice and the European Network of Centres for Pharmacoepidemiology and Pharmacovigilance. Informed consent was not required because the EPSS relied on routine pharmacovigilance and voluntary spontaneous reporting.

### 2.2 Objectives

The primary objective for each vaccine was to estimate the vaccinee RR of suspected ADRs occurring ≤7 days post‐vaccination in the overall population.

A secondary objective was to estimate the vaccinee RR occurring ≤7 days post‐vaccination according to pre‐defined age groups (≥6 months to <6 years, ≥6 to <13 years, ≥13 to <18 years, ≥18 to ≤65 years and >65 years) with SD‐IIV4 vaccine in Finland. For both vaccines, a secondary objective was to estimate the vaccinee RR for serious suspected ADRs over the entire follow‐up period.

A further secondary objective was to compare the vaccinee RR of suspected ADRs observed during the current EPSS with the established reactogenicity profile. In Germany, as this is was first season of EPSS for HD‐IIV4 vaccine, ADRs listed in the Summary of Product Characteristics (SmPC) were used for reference.^8^ For SD‐IIV4 vaccine in Finland, rates were compared with the previous NH influenza season^13^ and with the SmPC.^11^

### 2.3 Vaccines and vaccination

In Germany, participants received HD‐IIV4 vaccine (Efluelda®, Sanofi) containing 60 μg haemagglutinin antigen (HA) per influenza strain. In Finland, participants received SD‐IIV4 vaccine (Vaxigrip Tetra®, Sanofi) containing 15 μg HA per strain. Influenza strains were those recommended for the NH 2021/22 influenza season: A/Victoria/2570/2019 (H1N1)pdm09‐like virus; A/Cambodia/e0826360/2020 (H3N2)‐like virus; B/Washington/02/2019 (B/Victoria lineage)‐like virus; and B/Phuket/3073/2013 (B/Yamagata lineage)‐like virus.

Both vaccines were administered intramuscularly.

### 2.4 Vaccination cards and EDC reporting

Vaccination cards (VC) were provided to the vaccinee or vaccinee's parents/legal guardians to facilitate data collection. A copy of the VC was retained at the site so that the details could be transcribed into the EDC system. HCPs informed each vaccinee or vaccinee's parents/legal guardians on the importance of reporting suspected ADRs, particularly those occurring within the first 7 days, and instructed them on how to use the EDC system (Viedoc 4.60), which was set up using vaccinee‐specific accounts, to report any ADRs directly in ‘real‐time’. Alternatively, vaccinees or vaccinees' parents/legal guardians could report ADRs by telephone to Sanofi Pharmacovigilance affiliates in Finland or Germany. All data were extracted from the pharmacovigilance database for statistical analyses.

### 2.5 Data collected

The medical dictionary for regulatory activities (MedDRA) was used to define ADRs by system organ class (SOC) and preferred term (PT). Vaccinees were asked to report the intensity of ADRs as mild, moderate or severe. Serious ADRs were defined as those that resulted in death or are life‐threatening, or that required inpatient hospitalization or the prolongation of existing hospitalization, or resulted in the persistence of significant disability or incapacity.

Adverse events of interest (AEIs) as identified by the pharmacovigilance risk assessment committee (PRAC) were analysed separately and classified as systemic reactions (fever [≥38°C], nausea, vomiting, malaise, headache, decreased appetite, myalgia and/or arthralgia, irritability/prolonged crying [aged <5 years]), injection‐site reactions (pain, erythema and swelling) and events indicative of allergic and hypersensitivity reactions, including rash and ocular symptoms.

ADRs identified as potential risks as defined in the risk management plan (RMP) were anaphylactic reaction, vasculitis, thrombocytopenia and nervous system disorders such as convulsions (including febrile), Guillain‐Barré syndrome, encephalitis/transverse myelitis and neuritis.

ADRs that did not qualify as AEIs or in the RMP were termed as ‘other ADRs’.

### 2.6 Statistical analysis

The EPSS current interim guidance for seasonal influenza vaccines in the EU (EMA/PRAC/222346/2014) requires the system to be able to detect ADRs considered to be common, that is, expected to occur with a RR of ≥1%.^1^ To meet this requirement, 1000 individuals vaccinated within 4 to 6 weeks were to be included across the participating sites in each country. This population size provides a > 99% probability of collecting ≥1 report of a common AE.

Descriptive statistics were used to summarize the data, including the vaccinee RR with associated two‐sided 95% CIs. The vaccinee RR was calculated using the number of vaccinees who reported at least one suspected ADR divided by the total number of vaccinees. The vaccinee RR 95% CIs were calculated using the Clopper‐Pearson exact CI. Data were summarized cumulatively by ADRs occurring ≤7 or >7 days post‐vaccination or missing, by intensity and by pre‐defined age group for SD‐IIV4 vaccine in Finland.

## 3 RESULTS

### 3.1 Vaccinees

For HD‐IIV4 vaccine in Germany, 903 VCs were distributed to adults (aged ≥60 years). For SD‐IIV4 vaccine in Finland, 1000 VCs were distributed. In Finland, the majority of the vaccinees (90.6%) were aged ≥18 to ≤65 years; 84 (8.4%) were aged >65 years; five (0.5%) were aged ≥13 to <18 years; and four (0.4%) were aged ≥6 to <13 years. No vaccinees were aged <6 years.

### 3.2 HD‐IIV4 in Germany: ADRs during ≤7 days post‐vaccination

A total of 17 vaccinees reported 53 ADRs in the ≤7 days post‐vaccination, with a vaccinee RR of 1.88% (95% CI: 1.10, 3.00) (Table 1). ADR duration (ADR onset to stop date) was known for 27 ADRs, of which 24 (88.9%) resolved on the same day as onset or the following day, and three (11.1%) resolved within the first 3 days.

**TABLE 1:** Overview of ADR reporting in Germany (HD‐IIV4) and Finland (SD‐IIV4)

	Vaccinees reporting ≥1 suspected ADR(s), *n*	Number of ADRs	Vaccinee RR, % (95% CI)
**Germany, VCs distributed**, *n* = 903			
During the entire follow‐up period^†^	19	65	2.10 (1.27, 3.27)
ADRs ≤7 days of vaccination	17	53	1.88 (1.10, 3.00)
AEIs ≤7 days of vaccination	17	41	1.88 (1.10, 3.00)
Other ADRs ≤7 days of vaccination	5	12	0.55 (0.18, 1.29)
**Finland, VCs distributed**, *n* = 1000			
All ages			
ADR during the follow‐up period^‡^	51	134	5.10 (3.82, 6.65)
ADR ≤ 7 days of vaccination	49	125	4.90 (3.65, 6.43)
AEIs ≤7 days of vaccination	47	96	4.70 (3.47, 6.20)
Other ADRs ≤7 days of vaccination	16	29	1.60 (0.92, 2.59)
Age groups			
Aged ≥6 months to <6 years	–	–	–
Aged ≥6–<13 years			
ADR ≤ 7 days of vaccination	0	0	0.00 (0.00, 60.24)
Aged ≥13–<18 years			
ADR ≤ 7 days of vaccination	1	1	20.00 (0.51, 71.64)
Aged ≥18–≤65 years			
ADR ≤ 7 days of vaccination	47	121	5.19 (3.84, 6.84)
Aged >65 years			
ADR ≤ 7 days of vaccination	1	3	1.19 (0.03, 6.46)

Abbreviations: ADR, adverse drug reaction; AEI, adverse events of special interest; HD, high‐dose; IIV4, quadrivalent inactivated split‐virion influenza vaccine; RR, reporting rate; SD, standard dose; VC, vaccine card; ‘–’, there were no vaccinees in this age group.

^†^ The time to onset was known for 53/65 ADRs, 41/47 AEIs and 12/18 ‘other’ ADRs.

^‡^ The time to onset was known for 126/134 ADRs, 97/97 AEIs and 29/37 ‘other’ ADRs.

All 17 vaccinees reported at least one AEI, with a total of 41 events reported ≤7 days post‐vaccination, with a vaccinee RR of 1.88% (95% CI: 1.10, 3.00). AEIs were most frequently injection‐site reaction, headache and myalgia (Table 2). There were 12 ‘other’ ADRs reported by five vaccinees that occurred ≤7 days post‐vaccination, with a vaccinee RR of 0.55% (95% CI: 0.18, 1.29) (Table 3). ‘Other’ ADRs were most frequently chills, fatigue and feeling hot.

**TABLE 2:** Reporting of AEIs in Germany (HD‐IIV4) and Finland (SD‐IIV4), by time of onset

	Germany VCs distributed, *n* = 903			Finland VCs distributed, *n* = 1000					
	≤7 days post‐vaccination^†^			≤7 days post‐vaccination			>7 days		
	*n*	nAEI	Vaccinee RR, % (95% CI)	*n*	nAEI	Vaccinee RR, % (95% CI)	*n*	nAEI	Vaccinee RR, % (95% CI)
Any AEI^‡^	17	41	1.88 (1.10, 3.00)	47	96	4.70 (3.47, 6.20)	1	1	0.10 (0.00, 0.56)
Injection‐site reaction	14	16	1.55 (0.85, 2.59)	37	48	3.70 (2.62, 5.06)	0	0	0.00 (0.00, 0.37)
Headache	6	6	0.66 (0.24, 1.44)	14	14	1.40 (0.77, 2.34)	0	0	0.00 (0.00, 0.37)
Myalgia	4	4	0.44 (0.12, 1.13)	12	12	1.20 (0.62, 2.09)	0	0	0.00 (0.00, 0.37)
Fever	4	4	0.44 (0.12, 1.13)	5	5	0.50 (0.16, 1.16)	0	0	0.00 (0.00, 0.37)
Arthralgia	4	4	0.44 (0.12, 1.13)	2	2	0.20 (0.02, 0.72)	0	0	0.00 (0.00, 0.37)
Malaise	3	3	0.33 (0.07, 0.97)	5	5	0.50 (0.16, 1.16)	0	0	0.00 (0.00, 0.37)
Decreased appetite	2	2	0.22 (0.03, 0.80)	1	1	0.10 (0.00, 0.56)	1	1	0.10 (0.00, 0.56)
Nausea	1	1	0.11 (0.00, 0.62)	3	3	0.30 (0.06, 0.87)	0	0	0.00 (0.00, 0.37)
Vomiting	1	1	0.11 (0.00, 0.62)	–	–	–	–	–	–
Events indicative of allergic and hypersensitivity reactions^§^	–	–	–	2	6	0.20 (0.02, 0.72)	0	0	0.00 (0.00, 0.37)

Abbreviations: ADR, adverse drug reaction; AEI, adverse events of special interest; CI, confidence interval; HD, high‐dose; IIV4, quadrivalent inactivated split‐virion influenza vaccine; n, number of vaccinees; nADR, number of ADRs; RR, reporting rate; SD, standard dose; VC, vaccine card; ‘–’not reported.

^†^ No AEIs were reported >7 days post‐vaccination.

^‡^ AEIs with known time of onset: 41/47 (Germany) and 97/97 (Finland).

^§^ Including rash and ocular symptoms.

**TABLE 3:** Reporting of ‘other’ ADRs in Germany (HD‐IIV4), by time of onset

	≤7 days post‐vaccination VC distributed *n* = 903			During follow‐up period VC distributed *n* = 903		
	*n*	nADRs^‡^	Vaccinee RR, % (95% CI)	*n*	nADRs^§^	Vaccinee RR, % (95% CI)
‘Other’ ADR	5	12	0.55 (0.18, 1.29)	7	18	0.78 (0.31, 1.59)
Chills	2	2	0.22 (0.03, 0.80)	4	4	0.44 (0.12, 1.13)
Fatigue	3	3	0.33 (0.07, 0.97)	4	4	0.44 (0.12, 1.13)
Feeling hot	2	2	0.22 (0.03,0.80)	2	2	0.22 (0.03, 0.80)
General physical health deterioration	1	1	0.11 (0.00, 0.62)	1	1	0.11 (0.00, 0.62)
Influenza like illness	1	1	0.11 (0.00, 0.62)	1	1	0.11 (0.00, 0.62)
Nasal congestion	1	1	0.11 (0.00, 0.62)	1	1	0.11 (0.00, 0.62)
Oropharyngeal pain	1	1	0.11 (0.00, 0.62)	1	1	0.11 (0.00, 0.62)
Cardiovascular disorders	0	0	0.00 (0.00, 0.41)	1	1	0.11 (0.00, 0.62)
Influenza	1	1	0.11 (0.00, 0.62)	1	1	0.11 (0.00, 0.62)
Heart rate increased	0	0	0.00 (0.00, 0.41)	1	1	0.11 (0.00, 0.62)
Hypertension	0	0	0.00 (0.00, 0.41)	1	1	0.11 (0.00, 0.62)

Abbreviations: ADR, adverse drug reaction; AEI, adverse events of special interest; CI, confidence interval; HD, high‐dose; IIV4, quadrivalent inactivated split‐virion influenza vaccine; n, number of vaccinees; nADR, number of ADRs; PT, preferred term; RR, reporting rate; VC, vaccine card.

^†^ Among 12 ‘other’ ADRs with time to onset known, none were reported >7 days post‐vaccination.

^‡^ Time to onset known.

^§^ Time to onset not known for five nADRs.

### 3.3 HD‐IIV4 in Germany: ADRs during follow‐up

Overall, during the follow‐up period (including those with missing time of onset), 19 vaccinees reported 65 ADRs, the mean number of ADRs per vaccinee was 3.4 (SD: 2.61) and the maximum number of ADRs reported by a single vaccinee was nine (Table 1). There were no serious AEIs or RMP ADRs reported.

Among all ADRs, 72.31% (47/65) were AEIs. Intensity was reported for 39 AEIs, of which 16 (11 vaccinees) were mild, 14 (six vaccinees) were moderate and nine (four vaccinees) were severe (Table 4). The severe ADRs were injection‐site reaction (*n* = 3; all, pain), headache (*n* = 3), arthralgia (*n* = 1), nausea (*n* = 1) and vomiting (*n* = 1).

**TABLE 4:** Reporting of AEIs and ‘other’ ADRs in Germany (HD‐IIV4) and Finland (SD‐IIV4), by intensity (during the entire follow‐up period)

	Intensity^†^								
	Mild			Moderate			Severe		
	*n*	nADR	Vaccinee RR, % (95% CI)	*n*	nADR	Vaccinee RR, % (95% CI)	*n*	nADR	Vaccinee RR, % (95% CI)
**Germany** **VCs distributed**, *n* = 903									
**Any AEI**	11	16	1.22 (0.61, 2.17)	6	14	0.66 (0.24, 1.44)	4	9	0.44 (0.12, 1.13)
Injection‐site reaction	8	9	0.89 (0.38, 1.74)	4	4	0.44 (0.12, 1.13)	3	3	0.33 (0.07, 0.97)
Headache	0	0	0.00 (0.00, 0.41)	3	3	0.33 (0.07, 0.97)	3	3	0.33 (0.07, 0.97)
Myalgia	2	2	0.22 (0.03, 0.80)	3	3	0.33 (0.07, 0.97)	0	0	0.00 (0.00, 0.41)
Fever	1	1	0.11 (0.00, 0.62)	0	0	0.00 (0.00, 0.41)	0	0	0.00 (0.00, 0.41)
Arthralgia	2	2	0.22 (0.03, 0.80)	1	1	0.11 (0.00, 0.62)	1	1	0.11 (0.00, 0.62)
Malaise	0	0	0.00 (0.00, 0.41)	3	3	0.33 (0.07, 0.97)	0	0	0.00 (0.00, 0.41)
Decreased appetite	2	2	0.22 (0.03, 0.80)	0	0	0.00 (0.00, 0.41)	0	0	0.00 (0.00, 0.41)
Nausea	0	0	0.00 (0.00, 0.41)	0	0	0.00 (0.00, 0.41)	1	1	0.11 (0.00, 0.62)
Vomiting	0	0	0.00 (0.00, 0.41)	0	0	0.00 (0.00, 0.41)	1	1	0.11 (0.00, 0.62)
**‘Other’ ADR**	1	1	0.11 (0.00, 0.62)	2	4	0.22 (0.03, 0.80)	2	7	0.22 (0.03, 0.80)
Chills	0	0	0.00 (0.00, 0.41)	1	1	0.11 (0.00, 0.62)	1	1	0.11 (0.00, 0.62)
Fatigue	1	1	0.11 (0.00, 0.62)	1	1	0.11 (0.00, 0.62)	1	1	0.11 (0.00, 0.62)
Feeling hot	0	0	0.00 (0.00, 0.41)	1	1	0.11 (0.00, 0.62)	1	1	0.11 (0.00, 0.62)
General physical health deterioration	0	0	0.00 (0.00, 0.41)	0	0	0.00 (0.00, 0.41)	1	1	0.11 (0.00, 0.62)
Influenza like illness	0	0	0.00 (0.00, 0.41)	0	0	0.00 (0.00, 0.41)	1	1	0.11 (0.00, 0.62)
Nasal congestion	0	0	0.00 (0.00, 0.41)	0	0	0.00 (0.00, 0.41)	1	1	0.11 (0.00, 0.62)
Oropharyngeal pain	0	0	0.00 (0.00, 0.41)	0	0	0.00 (0.00, 0.41)	1	1	0.11 (0.00, 0.62)
CV disease	0	0	0.00 (0.00, 0.41)	0	0	0.00 (0.00, 0.41)	0	0	0.00 (0.00, 0.41)
Influenza	0	0	0.00 (0.00, 0.41)	1	1	0.11 (0.00, 0.62)	0	0	0.00 (0.00, 0.41)
Heart rate increased	0	0	0.00 (0.00, 0.41)	0	0	0.00 (0.00, 0.41)	0	0	0.00 (0.00, 0.41)
Hypertension	0	0	0.00 (0.00, 0.41)	0	0	0.00 (0.00, 0.41)	0	0	0.00 (0.00, 0.41)
**Finland** **VCs distributed**, *n* = 1000									
**Any AEI**	33	42	3.30 (2.28, 4.60)	21	40	2.10 (1.30, 3.19)	1	3	0.10 (0.00, 0.56)
Injection‐site reaction	29	34	2.90 (1.95, 4.14)	9	11	0.90 (0.41, 1.70)	0	0	0.00 (0.00, 0.37)
Headache	2	2	0.20 (0.02, 0.72)	11	11	1.10 (0.55, 1.96)	1	1	0.10 (0.00, 0.56)
Myalgia	4	4	0.40 (0.11, 1.02)	6	6	0.60 (0.22, 1.30)	0	0	0.00 (0.00, 0.37)–
Events indicative of allergic and hypersensitivity reactions	1	1	0.10 (0.00, 0.56)	1	5	0.10 (0.00, 0.56)	0	0	0.00 (0.00, 0.37)
Malaise	1	1	0.10 (0.00, 0.56)	3	3	0.30 (0.06, 0.87)	0	0	0.00 (0.00, 0.37)
Arthralgia	0	0	0.00 (0.00, 0.37)	1	1	0.10 (0.00, 0.56)	1	1	0.10 (0.00, 0.56)
Decreased appetite	0	0	0.00 (0.00, 0.37)	0	0	0.00 (0.00, 0.37)	1	1	0.10 (0.00, 0.56)
Nausea	0	0	0.00 (0.00, 0.37)	3	3	0.30 (0.06, 0.87)	0	0	0.00 (0.00, 0.37)
**‘Other’ ADR**	7	8	0.70 (0.28, 144)	10	19	1.00 (0.48, 1.83)	1	1	0.10 (0.00, 0.56)
Fatigue	2	2	0.20 (0.02, 0.72)	3	3	0.30 (0.06, 0.87)	0	0	0.00 (0.00, 0.37)
Pyrexia	0	0	0.00 (0.00, 0.37)	2	2	0.20 (0.02, 0.72)	0	0	0.00 (0.00, 0.37)
Axillary pain	1	1	0.10 (0.00, 0.56)	1	1	0.10 (0.00, 0.56)	0	0	0.00 (0.00, 0.37)
Chills	0	0	0.00 (0.00, 0.37)	1	1	0.10 (0.00, 0.56)	0	0	0.00 (0.00, 0.37)
Feeling abnormal	0	0	0.00 (0.00, 0.37)	1	1	0.10 (0.00, 0.56)	0	0	0.00 (0.00, 0.37)
Back pain	0	0	0.00 (0.00, 0.37)	2	2	0.20 (0.02, 0.72)	1	1	0.10 (0.00, 0.56)
Musculoskeletal stiffness	0	0	0.00 (0.00, 0.37)	1	1	0.10 (0.00, 0.56)	0	0	0.00 (0.00, 0.37)
Pain in extremity	0	0	0.00 (0.00, 0.37)	1	1	0.10 (0.00, 0.56)	0	0	0.00 (0.00, 0.37)
Oropharyngeal pain	0	0	0.00 (0.00, 0.37)	3	3	0.30 (0.06, 0.87)	0	0	0.00 (0.00, 0.37)
Body temperature increased	2	2	0.20 (0.02, 0.72)	0	0	0.00 (0.00, 0.37)	0	0	0.00 (0.00, 0.37)
Blood urine present	0	0	0.00 (0.00, 0.37)	0	0	0.00 (0.00, 0.37)	0	0	0.00 (0.00, 0.37)
Lymphadenopathy	1	1	0.10 (0.00, 0.56)	1	1	0.10 (0.00, 0.56)	0	0	0.00 (0.00, 0.37)
Influenza	0	0	0.00 (0.00, 0.37)	1	1	0.10 (0.00, 0.56)	0	0	0.00 (0.00, 0.37)
Rhinitis	1	1	0.10 (0.00, 0.56)	0	0	0.00 (0.00, 0.37)	0	0	0.00 (0.00, 0.37)
Ocular hyperaemia	0	0	0.00 (0.00, 0.37)	1	1	0.10 (0.00, 0.56)	0	0	0.00 (0.00, 0.37)
Abdominal discomfort	0	0	0.00 (0.00, 0.37)	1	1	0.10 (0.00, 0.56)	0	0	0.00 (0.00, 0.37)
Sensory disturbance	0	0	0.00 (0.00, 0.37)	0	0	0.00 (0.00, 0.37)	0	0	0.00 (0.00, 0.37)
Pruritis	1	1	0.10 (0.00, 0.56)	0	0	0.00 (0.00, 0.37)	0	0	0.00 (0.00, 0.37)

Abbreviations: ADR, adverse drug reaction; AEI, adverse events of special interest; CI, confidence interval; HD, high‐dose; IIV4, quadrivalent inactivated split‐virion influenza vaccine; n, number of vaccinees; nADR, number of ADRs; RR, reporting rate; SD, standard dose; VC, vaccine card.

^†^ AEIs with known intensity: 39/47 (Germany) and 85/97 (Finland).

During follow‐up, among all ADRs, 27.69% (18/65) were ‘other’ ADRs, of which 12 events reported by five vaccinees occurred ≤7 days post‐vaccination (Table 3).

Overall, intensity was reported for 12 ‘other’ ADRs, including one, four, and seven, that were mild, moderate and severe, respectively (Table 4). Among the seven severe ‘other’ ADRs, reported by two vaccinees, there was one event each of chills, fatigue, feeling hot, general physical health deterioration, influenza like illness, nasal congestion and oropharyngeal pain (Table 4).

### 3.4 SD‐IIV4 in Finland: ADRs during ≤7 days post‐vaccination

A total of 49 vaccinees reported 125 ADRs in the ≤7 days post‐vaccination, with a vaccinee RR of 4.90% (95% CI: 3.65, 6.43) (Table 1). The mean time to ADR onset was 1.1 days (SD: 1.56). A total of 96 AEIs, reported by 47 vaccinees, occurred ≤7 days post‐vaccination, with a vaccinee RR of 4.70% (95% CI: 3.47, 6.20). AEIs were most frequently injection‐site reaction, headache and myalgia (Table 2).

There were 29 ‘other’ ADRs, with a vaccinee RR of 1.60% (95% CI: 0.92, 2.59) (Table 5). ‘Other’ ADRs were most frequently fatigue, pyrexia, back pain and oropharyngeal pain (Table 4 and Table 5).

**TABLE 5:** Reporting of ‘other’ ADRs in Finland (SD‐IIV4), by time of onset

	≤7 days of vaccination			During follow‐up period		
	VCs distributed, *n* = 1000			VCs distributed, *n* = 1000		
	*n*	nADRs^†^	Vaccinee RR, % (95% CI)	*n*	nADRs^‡^	Vaccinee RR, % (95% CI)
**‘Other’ ADRs**	16	29	1.60 (0.92, 2.59)	20	37	2.00 (1.23, 3.07)
Fatigue	5	5	0.50 (0.16, 1.16)	6	6	0.60 (0.22, 1.30)
Pyrexia	3	3	0.30 (0.06, 0.87)	4	4	0.40 (0.11, 1.02)
Axillary pain	2	2	0.20 (0.02, 0.72)	2	2	0.20 (0.02, 0.72)
Chills	1	1	0.10 (0.00, 0.56)	1	1	0.10 (0.00, 0.56)
Feeling abnormal	1	1	0.10 (0.00, 0.56)	1	1	0.10 (0.00, 0.56)
Back pain	3	3	0.30 (0.06, 0.87)	3	3	0.30 (0.06, 0.87)
Musculoskeletal stiffness	1	1	0.10 (0.00, 0.56)	1	1	0.10 (0.00, 0.56)
Pain in extremity	1	1	0.10 (0.00, 0.56)	1	1	0.10 (0.00, 0.56)
Body temperature increased	1	1	0.10 (0.00, 0.56)	2	2	0.20 (0.02, 0.72)
Blood urine present	1	1	0.10 (0.00, 0.56)	1	1	0.10 (0.00, 0.56)
Oropharyngeal pain	3	3	0.30 (0.06, 0.87)	4	4	0.40 (0.11, 1.02)
Lymphadenopathy	2	2	0.20 (0.02, 0.72)	2	2	0.20 (0.02, 0.72)
Influenza	1	1	0.10 (0.00, 0.56)	1	1	0.10 (0.00, 0.56)
Rhinitis	1	1	0.10 (0.00, 0.56)	1	1	0.10 (0.00, 0.56)
Ocular hyperaemia	1	1	0.10 (0.00, 0.56)	1	1	0.10 (0.00, 0.56)
Sensory disturbance	0	0	0.00 (0.00, 0.37)	1	1	0.10 (0.00, 0.56)
Pruritis	1	1	0.10 (0.00, 0.56)	1	1	0.10 (0.00, 0.56)

Abbreviations: ADR, adverse drug reaction; AEI, adverse events of special interest; CI, confidence interval; IIV4, quadrivalent inactivated split‐virion influenza vaccine; n, number of vaccinees; nADR, number of ADRs; PT, preferred term; RR, reporting rate; SD, standard dose; VC, vaccine card.

^†^ ‘Other’ ADRs with known time to onset: 29/37.

^‡^ Including ‘other’ ADRs with unknown time to onset.

No ADRs were reported by vaccinees aged ≥6 to ≤13 years (VCs distributed, *n* = 4), and one vaccinee aged ≥13 to ≤18 years reported one ADR (Table 1). Among those aged ≥18 to ≤65 years, 47 vaccinees reported 121 ADRs, with a vaccinee RR of 5.19% (95% CI: 3.84, 6.84), and among those aged >65 years, one vaccinee reported three ADRs, with a vaccinee RR of 1.19% (95% CI: 0.03, 6.46).

### 3.5 SD‐IIV4 in Finland: ADRs during follow‐up

In Finland, overall, 51 vaccinees reported 134 ADRs (Table 1). The mean number of ADRs per vaccinee was 2.6 (SD: 2.48), and the maximum number of ADRs reported by a single vaccinee was 13. The majority (78.6% [99/126]) of ADRs occurred on the same day or the day after vaccination, 8.7% (11/126 ADRs) within 2 days, 11.9% (15/126 ADRs) within 6 days of vaccination and one, 9 days after vaccination. ADR duration was known for 91/134 ADRs, of which 7.7% (7/91) resolved on the same day as onset and 80.2% (73/134) resolved ≤2 days of onset. There were no serious ADRs reported or RMP ADRs.

Most of the ADRs were of moderate or mild intensity (Table 5), although the intensity was not reported for 12 vaccinees (21 ADRs). One vaccinee reported four ADRs of severe intensity (4/134, 3.0%), with a vaccinee RR of 0.10% (95% CI: 0.00, 0.56). The severe ADRs were headache, arthralgia, decreased appetite and back pain.

Among all ADRs, 72.4% (97/134) were AEIs, with a vaccinee RR of 4.70% (95% CI: 3.47, 6.20). All but four of the vaccinees (92.16%) who reported ≥1 suspected ADR, reported ≥1 AEI (Table 2). The distribution of AEIs by age group is shown in Supporting information, Table S1.

Overall, a total of 20 vaccinees reported 37 ‘other’ ADRs, with a vaccinee RR of 2.00% (95% CI: 1.23, 3.07). Except for the SOC ‘General disorders and administration site conditions’ with 13 vaccinees reporting 16 ADRs (PTs: asthenia and fatigue), the SOCs for all the ‘other’ ADRs had ≤5 events reported. Two vaccinees aged >65 years reported five ‘other’ ADRs (PTs: oropharyngeal pain and rhinorrhoea and abdominal discomfort), and the remaining 32 ‘other’ ADRs were reported by 18 vaccinees aged ≥18 years to ≤65 years (Supporting information, Table S2).

### 3.6 Comparison with previous experience

#### 3.6.1 HD‐IIV4 in Germany

With HD‐IIV4, the vaccinee RRs for AEIs with onset ≤7 days post‐vaccination with the PTs of vaccination site pain (1.55%; 95% CI: 0.85, 2.59), erythema (0.11%; 95% CI: 0.00, 0.62), vaccination site erythema (0.11%; 95% CI: 0.00, 0.62), headache (0.66%; 95% CI: 0.24, 1.44), myalgia (0.66%; 95% CI: 0.24, 1.44) and malaise (0.33%; 95% CI: 0.07, 0.97) were substantially lower than those in the SmPC, which lists these events as very common (≥10%).^8^

ADRs reported in Germany occurring ≤7 days post‐vaccination, and which were not listed in the SmPC, were vaccination site warmth (one ADR), decreased appetite (two ADRs), influenzalike illness (one ADR), nasal congestion (one ADR) and general physical health deterioration (one ADR). ADRs reported in Germany occurring >7 days post‐vaccination that were not listed in the SmPC were heart rate increased (one ADR), cardiovascular disorder (one ADR) and hypertension (one ADR).

#### 3.6.2 SD‐IIV4 in Finland

With SD‐IIV4, the vaccinee RRs in the current 2021/22 influenza season were higher than in the 2020/21 influenza season (above upper limit of previous season's CI) for overall ADRs, ADRs occurring ≤7 days post‐vaccination, and AEIs.^13^ For the ADRs that occurred ≤7 days post‐vaccination, for current and previous influenza season, the vaccinee RRs were 4.90% (95% CI: 3.65, 6.43) and 2.78% (95% CI: 1.85, 3.99), respectively. For AEIs that occurred ≤7 days post‐vaccination, for current and previous influenza season, the vaccinee RR was 4.70% (95% CI: 3.47, 6.20) and 2.78% (95% CI: 1.85, 3.99).

All ADRs were non‐serious in both seasons. There were four severe ADRs reported in the current season versus two severe ADRs in the previous season. There were 21 ADRs that were reported in the current season and were not listed in the SmPC and nor reported in the previous season^11^, ^13^: those occurring ≤7 days post‐vaccination were (three events each) decreased appetite, mass, neck pain, rash macular, pain, sensitive skin, urticaria and back pain and (one event each) abdominal discomfort, axillary pain, blood urine present, feeling abnormal, musculoskeletal stiffness, ocular hyperaemia, pain in extremity and rhinitis.

## 4 DISCUSSION

The results of the EPSS conducted in Germany and Finland during the NH 2020/21 influenza season were consistent with the established safety profiles of the IIV4 vaccines. Among 903 VCs distributed to HD‐IIV4 vaccine recipients in Germany, during ≤7 days post‐vaccination, 17 vaccinees reported 53 ADRs at a vaccinee RR of 1.88% (95% CI: 1.10, 3.00). Among 1000 VCs distributed to recipients of SD‐IIV4 vaccine in Finland, during ≤7 days post‐vaccination, 49 vaccinees reported 125 ADRs with a vaccine RR of 4.90% (95% CI: 3.65, 6.43). None of the ADRs were serious.

The German Standing Committee on Vaccinations recommends HD‐IIV4 for people aged ≥60 years.^14^ In our EPSS in Germany, VCs were distributed at 10 participating sites among people aged ≥60 years receiving HD‐IIV4 in October 2021. The NH 2021/22 influenza season in Germany was the first use of the HD‐IIV4 vaccine, so clinical trial and post‐marketing data were used as a reference for the established safety profile of the vaccine. Among the PTs reported in Germany, seven were not listed in the SmPC,^8^ of which influenzalike illness, nasal congestion and general physical health deterioration occurred ≤7 days post‐vaccination, and increased heart rate, cardiovascular disorder and hypertension occurred >7 days post‐vaccination. In addition, the PTs pain in the extremity, arthralgia and feeling hot had vaccinee RRs of 0.11% to 0.44%, compared with ≥0.01% to <0.1% as listed in the SmPC.^8^ However, several of the ADRs reported occurred at a lower or similar rate as stated in the SmPC, and overall with HD‐IIV4 in Germany, there was no reporting pattern by type, frequency or severity of ADRs.

In Finland, the national immunization programme includes SD‐IIV4 vaccination for people aged ≥65 years, children aged 6 months to 6 years, pregnant women, people with comorbid conditions, HCPs, people in contact with people who are vulnerable to severe influenza and people starting military service.^15^ Although SD‐IIV4 vaccine was supplied by the Finnish National Institute for Health and Welfare during the 2021/22 influenza season, a LAIV4 nasal spray was also available for children aged 24 months to 6 years. In the EPSS, among 1000 VCs distributed in Finland, none of the vaccinees were children aged ≤6 years, and most were adults aged 18 to 65 years (90.6%) or aged >65 years (8.4%). The lack of representation of paediatric and elderly vaccinees in this study may be due to the private clinics included here implementing local workforce immunization campaigns outside of the national immunization programme, and that children and the elderly were eligible for free vaccination at public healthcare sites and so less likely to visit these private clinics.

In the NH 2021/22 influenza season EPSS for SD‐IIV4 in Finland, the vaccinee RR for ADRs occurring ≥7 days post‐vaccination was 4.90% (95% CI: 3.65, 6.43), compared with 2.78% (95% CI: 1.85, 3.99) in the 2020/21 season^13^ and 4.05% (95% CI: 2.79, 5.31) in 2019/21 season,^4^ all included individuals ≥6 months. The slightly lower RR observed in the 2020/21 season EPSS compared with the years before and after, were likely due to the impact and restrictions due to the COVID‐19 pandemic. Overall, there were 21 ADRs reported that were not listed in the SmPC and not reported during the previous season, including eight PTs, which were each reported three times ≥7 days post‐vaccination: decreased appetite, mass, neck pain, rash macular, pain, sensitive skin, urticaria and back pain.^11^ In the current season in Finland, although vaccination site pain was reported with a higher frequency than in the previous season, this PT is listed as very common in the SmPC.^11^

In October 2021, the WHO issued recommendations for the coadministration of influenza vaccines and COVID‐19 vaccines during the 2021/22 influenza season in Europe.^16^ In Germany, vaccinees could request coadministration of a COVID‐19 vaccination and were asked to report such, and while none of the vaccinees in the EPSS reported this coadministration in the EDC system, it is estimated that 10% of HD‐IIV4 recipients in this study may have received a COVID‐19 vaccine on the same day. In Finland, although none of the study sites coadministered COVID‐19 vaccine with SD‐IIV4, COVID‐19 vaccination was available to the vaccinees at vaccination centres outside of this study. Two participants in the Finland EPSS reported COVID‐19 vaccine as a ‘concomitant medication’ and thus may have received COVID‐19 vaccination on the same day as the study vaccine. As a result, these vaccinees may not have been able to discern the causality of the symptoms experienced, leading to the reporting of all experienced symptoms.

The choice of country for the current SD‐IIV4 vaccine EPSS was based on previous experience of surveillance in Finland, thus enabling the use of existing collaborations and resources, as well as the potential for comparison of RR across years in the same country. Over the past four seasons, data collection methods have changed, and the EPSS results from consecutive years in Finland suggest that the reporting method used has an effect on the ADR RR. In the 2018/19 season EPSS in Finland, using email reporting, the 7‐day ADR rate was 5.3% (95% CI: 3.9, 6.7),^15^ and in the 2019/20 season, using pre‐paid envelopes and telephone reporting, but not email, the 7‐day ADR rate was 12.5% (95% CI: 10.4, 14.7).^17^ Using reporting via the EDC system and telephone, in the 2020/21 season EPSS in Finland, the 7‐day ADR rate was 7.84% (95% CI: 6.25, 9.67),^13^ and in the current 2021/22 season, the 7‐day ADR rate was 12.50% (95% CI: 10.51, 14.71) (data not shown). Notwithstanding the effect of the pandemic on RR in the 2020/21 influenza season, the EPSS results for SD‐IIV4 vaccine in Finland suggest that EDC/telephone or paper/telephone reporting both capture more events than email reporting.^13^, ^17^ Using the EDC system, spontaneous reporting was stimulated by HCPs informing vaccinees about the importance of reporting ADRs, which could be done directly using the EDC system. Furthermore, the customized reporting form with pre‐defined ADRs and focused questions enabled the collection of accurate information for case processing and follow‐up by local pharmacovigilance staff.

The main limitation of the study was that all ADRs were self‐reported by vaccinees or their legal guardians and were not medically confirmed. To avoid bias in the collection of safety data, there was no possibility for follow‐up with individuals at the site level.

## 5 CONCLUSIONS

The NH 2021/22 EPSS results for HD‐IIV4 vaccine in Germany and SD‐IIV4 vaccine in Finland did not suggest any clinically significant change in safety beyond what is known or expected for IIV4s. Using EDC reporting with telephone back‐up, the reactogenicity profiles of the vaccines were consistent with published rates of spontaneous ADR reports with other seasonal influenza vaccines, ranging from 20 to 90 reports per 1,000,000 people vaccinated.^7^, ^18^, ^19^, ^20^, ^21^

## CONFLICTS OF INTEREST

SGB, SW, CM, OS and AS are employees of Sanofi and may hold shares and/or stock options in the company.

## AUTHOR CONTRIBUTIONS

**Sonja Gandhi‐Banga:** Conceptualization; writing‐review and editing. **Sophie Wague:** Conceptualization; writing‐review and editing. **Anju Shrestha:** Conceptualization; writing‐review and editing. **Olga Syrkina:** Conceptualization; writing‐review and editing. **Oxana Talanova:** Conceptualization; writing‐review and editing. **Markku Nissila:** Investigation; writing‐review and editing. **Karl Stuff:** Investigation; writing‐review and editing. **Celine Monfredo:** Conceptualization; data curation; formal analysis; writing‐review and editing.

### PEER REVIEW

The peer review history for this article is available at https://publons.com/publon/10.1111/irv.13071.

## Supporting information




**Table S1.** Reporting of AEIs occurring ≤7 days post‐vaccination (SD‐IIV4) by age‐group in Finland
**Table S2.** Reporting of ‘other’ ADRs occurring ≤7 days post vaccination (SD‐IIV4 vaccine) in Finland by age group

## Data Availability

The datasets generated and/or analysed during the current study, including the raw data, are not publicly available in order to safeguard the privacy of participants and the confidentiality and protection of their data, as well as protect commercially sensitive information. Qualified researchers may request access to patient level data and related study documents including the clinical study report, study protocol with any amendments, blank case report form, statistical analysis plan and dataset specifications. Patient level data will be anonymized, and study documents will be redacted to protect the privacy of our trial participants. Further details on Sanofi's data sharing criteria, including required permissions to access the data, eligible studies and process for requesting access can be found at: https://www.vivli.org/.
