# Supplementary material for: Enhanced passive safety surveillance of high‐dose and standard‐dose quadrivalent inactivated split‐virion influenza vaccines in Germany and Finland during the influenza season 2021/22
Source: Influenza Other Respir Viruses. 2022 Nov 29;17(1):e13071. doi: 10.1111/irv.13071 (PMC9835399; doi:10.1111/irv.13071)
Supplement: Supplementary file 1 — Table S1. Reporting of AEIs occurring ≤7 days post‐vaccination (SD‐IIV4) by age‐group in Finland Table S2. Reporting of ‘other’ ADRs occurring ≤7 days post vaccination (SD‐IIV4 vaccine) in Finland by age group [file IRV-17-e13071-s001.docx]

**Supplements**

**Supplement 1. Reporting of AEIs occurring ≤7 days post-vaccination (SD-IIV4) by age-group in Finland**

|  | **Aged ≥13 – <18 years**  **VC distributed, n=5** | | | **Aged ≥18 – ≤65 years**  **VC distributed, n=906** | | | **Aged >65 years**  **VC distributed, n=84** | | |
| --- | --- | --- | --- | --- | --- | --- | --- | --- | --- |
|  | **n** | **nADRs** | **Vaccinee RR, % (95% CI)** | **n** | **nADRs** | **Vaccinee RR, % (95% CI)** | **n** | **nADRs** | **Vaccinee RR, % (95% CI)** |
| All AEIs ≤7 days post-vaccination | 1 | 1 | 20.00 (0.51, 71.64) | 45 | 93 | 4.97 (3.65, 6.59) | 1 | 2 | 1.19 (0.03, 6.46) |
| Injection site reaction | 1 | 1 | 20.00 (0.51, 71.64) | 36 | 47 | 3.97 (2.80, 5.46) | 0 | 0 | – |
| Headache | 0 | 0 | – | 13 | 13 | 1.43 (0.77, 2.44) | 1 | 1 | 1.19 (0.03, 6.46) |
| Myalgia | 0 | 0 | – | 12 | 12 | 1.32 (0.69, 2.30) | 0 | 0 | – |
| Fever | 0 | 0 | – | 5 | 5 | 0.55 (0.18, 1.28) | 0 | 0 | – |
| Malaise | 0 | 0 | – | 5 | 5 | 0.55 (0.18, 1.28) | 0 | 0 | – |
| Arthralgia | 0 | 0 | – | 2 | 2 | 0.22 (0.03, 0.80) | 0 | 0 | – |
| Decreased appetite | 0 | 0 | – | 1 | 1 | 0.11 (0.00, 0.61) | 0 | 0 | – |
| Nausea | 0 | 0 | – | 2 | 2 | 0.22 (0.03, 0.80) | 1 | 1 | 1.19 (0.03, 6.46) |
| Events indicative of allergic and hypersensitivity reactions^†^ | 0 | 0 | – | 2 | 6 | 0.22 (0.03, 0.80) | 0 | 0 | – |

ADR, adverse drug reaction; AEI, adverse events of special interest; CI, confidence interval; n, number of vaccinees; nADR, number of ADRs; RR, reporting rate; SOC, system organ class; VC, vaccination card

^†^Rash and ocular symptoms

No VCs were distributed in children aged 6 months–<6 years; and no ADRs were reported among children aged ≥6 – <13 years (VCs distributed, n=4)

Time to onset was not known for 4/32 AEIs in the >65 years group

**Supplement 2: Reporting of ‘other’ ADRs occurring ≤7 days post vaccination (SD-IIV4 vaccine) in Finland by age group**

|  | **Aged ≥18 – ≤65 years**  **VC, n=906** | | | **Aged >65 years**  **VC, n=84** | | |
| --- | --- | --- | --- | --- | --- | --- |
|  | **n** | **nADRs** | **Vaccinee RR, % (95% CI)** | **n** | **nADRs** | **Vaccinee RR, % (95% CI)** |
| ‘Other’ ADRs occurring ≤7 days post vaccination | 15 | 28 | 1.66 (0.93, 2.72) | 1 | 1 | 1.19 (0.03, 6.46) |
| Fatigue | 5 | 5 | 0.55 (0.18,1.28) | 0 | 0 | 0.00 (0.00, 4.30) |
| Pyrexia | 3 | 3 | 0.33 (0.07, 0.96) | 0 | 0 | – |
| Axillary pain | 2 | 2 | 0.22 (0.03, 0.80) | 0 | 0 | – |
| Chills | 1 | 1 | 0.11 (0.00, 0.61) | 0 | 0 | – |
| Feeling abnormal | 1 | 1 | 0.11 (0.00, 0.61) | 0 | 0 | – |
| Back pain | 3 | 3 | 0.33 (0.07, 0.96) | 0 | 0 | – |
| Musculoskeletal stiffness | 1 | 1 | 0.11 (0.00, 0.61) | 0 | 0 | – |
| Pain in extremity | 1 | 1 | 0.11 (0.00, 0.61) | 0 | 0 | – |
| Lymphadenopathy | 2 | 2 | 0.22 (0.03, 0.80) | 0 | 0 | – |
| Body temperature increased | 1 | 1 | 0.11 (0.00, 0.61) | 0 | 0 | – |
| Blood urine present | 1 | 1 | 0.11 (0.00, 0.61) | 0 | 0 | – |
| Oropharyngeal pain | 3 | 3 | 0.33 (0.07, 0.96) | 0 | 0 | 0.00 (0.00, 4.30) |
| Influenza | 1 | 1 | 0.11 (0.00, 0.61) | 0 | 0 | – |
| Rhinitis | 1 | 1 | 0.11 (0.00, 0.61) | 0 | 0 | – |
| Ocular hyperaemia | 1 | 1 | 0.11 (0.00, 0.61) | 0 | 0 | – |
| Abdominal discomfort | 0 | 0 | – | 1 | 1 | 1.19 (0.03, 6.46) |
| Pruritis | 1 | 1 | 0.11 (0.00, 0.61) | 0 | 0 | – |

ADR, adverse drug reaction; AEI, adverse events of special interest; CI, confidence interval; n, number of vaccinees; nADR, number of ADRs; PT, preferred Term; RR, reporting rate; VC, vaccination card

No VCs were distributed in children aged 6 months–<6 years.

No ‘other’ ADRs were reported among children aged ≥6 – <13 years (VCs distributed, n=4), or among children aged ≥13–<18 years (VCs distributed, n=5)

Time to onset was not known for 4/32 ‘other’ ADRs in the ≥18 – ≤65 years group

Time to onset was not known for 4/5 ‘other’ ADRs in the aged >65 years group
